# ^123^I-BMIPP, a Radiopharmaceutical for Myocardial Fatty Acid Metabolism Scintigraphy, Could Be Utilized in Bacterial Infection Imaging

**DOI:** 10.3390/pharmaceutics14051008

**Published:** 2022-05-07

**Authors:** Yuka Muranaka, Asuka Mizutani, Masato Kobayashi, Koya Nakamoto, Miki Matsue, Fumika Takagi, Kenichi Okazaki, Kodai Nishi, Kana Yamazaki, Ryuichi Nishii, Naoto Shikano, Shigefumi Okamoto, Hideki Maki, Keiichi Kawai

**Affiliations:** 1 Division of Health Sciences, Graduate School of Medical Sciences, Kanazawa University, 5-11-80 Kodatsuno, Kanazawa 920-0942, Japan; yukarisa93@stu.kanazawa-u.ac.jp (Y.M.); kou.nakamoto@sage.ocn.ne.jp (K.N.); 2 Faculty of Health Sciences, Institute of Medical, Pharmaceutical and Health Sciences, Kanazawa University, 5-11-80 Kodatsuno, Kanazawa 920-0942, Japan; mizutani.a@staff.kanazawa-u.ac.jp (A.M.); kobayasi@mhs.mp.kanazawa-u.ac.jp (M.K.); sokamoto@mhs.mp.kanazawa-u.ac.jp (S.O.); 3 Ishikawa Prefectural Institute of Public Health and Environmental Science, 1-11, Taiyogaoka, Kanazawa 920-1154, Japan; mmiikkii_0804@pref.ishikawa.lg.jp; 4 Laboratory for Drug Discovery & Disease Research, Shionogi & Co., Ltd., 3-1-1, Futaba-cho, Toyonaka 561-0825, Japan; fumika.takagi@shionogi.co.jp (F.T.); kenichi.okazaki@shionogi.co.jp (K.O.); hideki.maki@shionogi.co.jp (H.M.); 5 Department of Radioisotope Medicine, Atomic Bomb Disease Institute, Nagasaki University, 1-12-4 Sakamoto, Nagasaki 852-8523, Japan; koudai@nagasaki-u.ac.jp; 6 Department of Molecular Imaging and Theranostics, Institute for Quantum Medical Science, Quantum Life and Medical Science Directorate, National Institutes for Quantum Science and Technology, 4-9-1 Anagawa, Inage, Chiba 263-8555, Japan; yamazaki.kana@qst.go.jp (K.Y.); nishii.ryuichi@qst.go.jp (R.N.); 7 Department of Radiological Sciences, Ibaraki Prefectural University of Health Sciences, 4669-2 Ami, Inashiki 300-0394, Japan; sikano@ipu.ac.jp; 8 Advanced Health Care Science Research Unit, Innovative Integrated Bio-Research Core Institute for Frontier Science Initiative, Kanazawa University, 5-11-80 Kodatsuno, Kanazawa 920-0942, Japan; 9 Biomedical Imaging Research Center, University of Fukui, 23-3 Matsuoka-shimoaizuki, Eiheiji-cho, Yoshida-gun, Fukui 910-1193, Japan

**Keywords:** ^123^I-BMIPP, SPECT, nuclear medicine imaging, bacterial infection, bacterial imaging

## Abstract

In this study, we evaluated the use of 15-(4-^123^I-iodophenyl)-3(R,S)-methylpentadecanoic acid (^123^I-BMIPP) to visualize fatty acid metabolism in bacteria for bacterial infection imaging. We found that ^123^I-BMIPP, which is used for fatty acid metabolism scintigraphy in Japan, accumulated markedly in *Escherichia coli* EC-14 similar to ^18^F-FDG, which has previously been studied for bacterial imaging. To elucidate the underlying mechanism, we evaluated changes in ^123^I-BMIPP accumulation under low-temperature conditions and in the presence of a CD36 inhibitor. The uptake of ^123^I-BMIPP by EC-14 was mediated via the CD36-like fatty-acid-transporting membrane protein and accumulated by fatty acid metabolism. In model mice infected with EC-14, the biological distribution and whole-body imaging were assessed using ^123^I-BMIPP and ^18^F-FDG. The ^123^I-BMIPP biodistribution study showed that, 8 h after infection, the ratio of ^123^I-BMIPP accumulated in infected muscle to that in control muscle was 1.31 at 60 min after ^123^I-BMIPP injection. In whole-body imaging 1.5 h after ^123^I-BMIPP administration and 9.5 h after infection, infected muscle exhibited a 1.33-times higher contrast than non-infected muscle. Thus, ^123^I-BMIPP shows potential for visualizing fatty acid metabolism of bacteria for imaging bacterial infections.

## 1. Introduction

Bacteria exhibiting antimicrobial resistance (AMR), such as fluoroquinolone-resistant *Escherichia coli* and penicillin-resistant *Streptococcus pneumoniae*, have emerged due to the inappropriate use of antimicrobial agents [1,2,3]. It is predicted that, as the number of bacteria resistant to existing antimicrobial agents increases, conventional treatment methods will no longer be applicable, and treatment will thus become more difficult. The World Health Organization has advocated the development of international AMR countermeasure action plans detailing the investigation and monitoring of AMR trends, the appropriate use of antimicrobial agents, and research and development strategies for new antimicrobial agents [4,5].

With the increasing awareness of the seriousness of infectious diseases involving AMR, molecular imaging techniques have attracted attention for bacterial infection imaging. Nuclear medicine imaging is particularly useful as a new non-invasive diagnostic method that can be used to identify the site of an infection and follow up on the effects of treatment [6,7]. Various radiopharmaceuticals have been investigated for bacterial infection imaging. The development and application of radiopharmaceuticals, such as ^99m^Tc-based derivatives, ^99m^Tc-labeled antibiotics, ^11^C-labeled D-amino acids, and 2-deoxy-2-[^18^F]fluoro-d-glucose (^18^F-FDG), which target components of the bacterial cell wall, are still in progress [6,8]. These compounds include not only carbon sources (D-glucose), nitrogen sources (amino acids), and minerals but also fatty acids, which are essential for bacterial growth [9,10]. However, to date, no radiopharmaceutical for bacterial infection imaging that targets fatty acid metabolism has been developed. We examined the use of 15-(4-^123^I-iodophenyl)-3(R,S)-methylpentadecanoic acid (^123^I-BMIPP), a radiopharmaceutical commonly used in single-photon emission computed tomography (SPECT) myocardial fatty acid metabolism scintigraphy in Japan, for bacterial imaging in comparison with ^18^F-FDG, which is already widely used in imaging.

## 2. Materials and Methods

^123^I-BMIPP was purchased from Nihon Medi-Physics Co., Ltd. (Tokyo, Japan). ^18^F-FDG was synthesized at the PET facility of Kanazawa University.

### 2.1. Bacterial Strain and Culture Conditions

*Escherichia coli* EC-14 was used as the clinical isolate strain. EC-14 was obtained from Shionogi & Co., Ltd. (Osaka, Japan). For pre-cultivation, stock EC-14 in 50% glycerol was mixed with THY medium consisting of Todd-Hewitt Broth (Becton, Dickinson and Co., Franklin Lakes, NJ, USA) and 0.2% yeast extract (Becton, Dickinson and Co.) at a ratio of 1:100. EC-14 was incubated at 37 °C for 12–14 h with shaking. Subsequently, EC-14 (1.2 × 10^8^ colony-forming units (CFU)/100 µL) was seeded in amino-acid-free Dulbecco’s Modified Eagle’s Medium (DMEM; Fujifilm Wako Pure Chemical Corporation, Osaka, Japan; 048-33575) and incubated at 37 °C with shaking. Bacterial protein was measured using a Pierce™ BCA Protein Assay kit (Thermo Fisher Scientific, Waltham, MA, USA; 23227).

### 2.2. Accumulation of Radiopharmaceuticals in E. coli EC-14

EC-14 (1.2 × 10^8^ CFU/100 µL) was seeded in 5 mL of amino-acid-free DMEM and incubated for 1, 3 and 6 h. After incubation, 37 kBq/10 µL of ^123^I-BMIPP and ^18^F-FDG was added and incubated for 5 min at 37 °C with shaking. For in vitro experiments using ^18^F-FDG, the concentration of glucose in the medium was adjusted to 0.1 mg/mL, since among the three glucose concentrations examined (4.5, 1.0 and 0.1 mg/mL), the highest accumulation of ^18^F-FDG was observed at a concentration of 0.1 mg/mL.

EC-14 was collected by centrifugation at 7000× *g* for 10 min at 4 °C and then washed three times with 5 mL of phosphate-buffered saline (PBS; Medical & Biological Laboratories Co., Ltd., Aichi, Japan). Samples were suspended in 1 mL of 0.1 M NaOH and measured using a gamma counter (AccuFLEX ARC-γ7010, Aloka Medical, Tokyo, Japan).

### 2.3. Accumulation of ^123^I-BMIPP in E. coli EC-14 under Low-Temperature Conditions

EC-14 (1.2 × 10^8^ CFU/100 µL) was seeded in 5 mL of amino-acid-free DMEM and incubated under ice-cold conditions at 4 °C for 3 h. After incubation, 37 kBq/10 µL of ^123^I-BMIPP was added to the solution, and they were incubated for 5 min at 4 °C with shaking. The radioactivity accumulated in the EC-14 was then measured as described in the Section 2.2. Accumulation of Radiopharmaceuticals in *E. coli* EC-14.

### 2.4. Accumulation of ^123^I-BMIPP in E. coli EC-14 in the Presence of a CD36 Inhibitor

EC-14 was cultured under the same conditions as described in Section 2.2. Accumulation of Radiopharmaceuticals in *E. coli* EC-14. After incubation at 37 °C, 37 kBq/50 µL of ^123^I-BMIPP and 50 µL of 1.0 mM sulfosuccinimidyl oleate (SSO, Cayman Chemical, Ann Arbor, MI, USA), an inhibitor of CD36, were mixed and added to the cell culture. CD36 binds long-chain fatty acids and promotes their transport into cells. The radioactivity accumulated in EC-14 was measured using the same method as described in the Section 2.2.

### 2.5. Mouse Model of E. coli EC-14 Infection

All experiments were conducted in accordance with the ethical standards of our university (Animal Care Committee of Kanazawa University, AP-183983) and with international standards for animal welfare and institutional guidelines. EC-14 was cultured in Luria–Bertani broth (Becton, Dickinson and Co., Franklin Lakes, NJ, USA) for 12 h and then seeded into new broth and incubated at 37 °C for 12–14 h with shaking. Subsequently, EC-14 was collected by centrifugation at 8000× *g* for 5 min at 4 °C and suspended for inoculation into mice. Male Jcl:ICR mice (*n* = 4) (4 weeks old; CLEA Japan, Tokyo, Japan) were purchased 7 days prior to the experiments and subjected to immunosuppression treatment with 150 mg/kg and 100 mg/kg of endoxan (Shionogi) at 4 days and 1 day prior to infection, respectively.

EC-14 (approximately 5 × 10^6^ CFU/100 µL) was injected into the muscle of the hind leg of mice under anesthesia. Mice were anesthetized using a mixture of butorphanol tartrate, midazolam, and medetomidine hydrochloride (Fujifilm Wako Pure Chemical Corp., Osaka, Japan). Mice were euthanized at 2, 6, 8 and 24 h after infection. Subsequently, the infected muscle was collected and homogenized in PBS. The number of CFU in the homogenized samples was determined by dilution in PBS and plating on agar medium.

### 2.6. Biological Distribution of ^123^I-BMIPP and ^18^F-FDG in E. coli EC-14 Infection Model Mice

EC-14 (approximately 5 × 10^6^ CFU/100 µL) was injected into the muscle of the hind leg of mice (*n* = 4) as described in the Section 2.5. At 2 and 8 h after infection, mice were injected intravenously with 370 kBq/110 µL of ^123^I-BMIPP and 10–17 MBq/90 µL of ^18^F-FDG. The mice injected with ^123^I-BMIPP and ^18^F-FDG were different individuals. Before administration, the mice were fasted for 4 h. Mice were euthanized at 15 and 60 min after administration, and blood, heart, lung, liver, kidney, infected perineal muscle, and contralateral non-infected perineal muscle (control muscle) tissues were collected. The collected organs were dissolved by adding 1.0 mL of solubilizer, Solvable (PerkinElmer, Waltham, MA, USA), and crushed with a disposable homogenizer, BioMasher^®^ (Nippi, Tokyo, Japan, 49118-71). These samples and initial ^123^I-BMIPP- and ^18^F-FDG-associated radioactivity were measured using a gamma counter (AccuFLEX ARC-8001; Hitachi Aloka Medical, Tokyo, Japan). In heart, lung and kidney, the radioactivity and weight of whole organs were measured. In liver, a part of the liver was cut out, and the radioactivity and weight were measured. Weight of whole liver was also measured, and the radioactivity of whole liver was calculated using radioactivity of a part of the liver. Data are reported as percent injected dose per gram of tissue (%ID/g).

### 2.7. Planar Imaging with ^123^I-BMIPP in E. coli EC-14 Infection Model Mice

At 8 h post-infection, ^123^I-BMIPP (18.5 MBq/300 µL/mouse) was injected into the tail vein of EC-14 infection model mice under lead shielding from head to bladder (*n* = 3). Imaging conditions were set as follows: matrix, 512 × 512 pixels; magnification, 100%. The mice were anesthetized, and a gamma camera (MiniCam, Inter Medical, Lübbecke, Germany) was used to image the mice.

Planar image was acquired for 5 min (1 frame) at 1.5 h after ^123^I-BMIPP administration and 9.5 h after EC-14 infection. Images were analyzed using AMIDE data analysis software (ver. 1.0.4).

### 2.8. Statistical Analysis

Data are presented as means and standard deviation and analyzed using the F-test and Student’s *t*-test. All analyses were conducted using GraphPad Prism 8 statistical software (GraphPad Software, Inc., La Jolla, CA, USA). A *p* value of less than 0.05 was considered indicative of a statistically significant difference.

## 3. Results

### 3.1. Accumulation of Radiopharmaceuticals in E. coli EC-14

**Figure 1:**
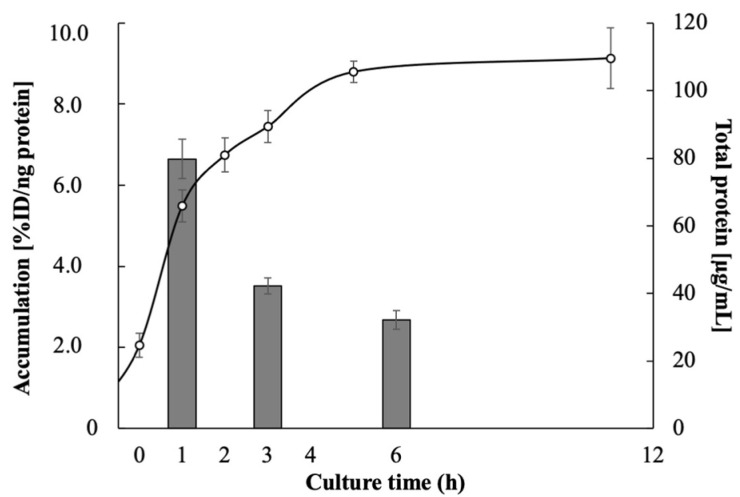
Accumulation of ^123^I-BMIPP in *E. coli* EC-14 5 min after addition. EC-14 was incubated in amino-acid-free DMEM for 1, 3 and 6 h. Accumulation of ^123^I-BMIPP is greatest 1 h after incubation, during the early logarithmic growth phase.

Table 1 shows the accumulation of ^123^I-BMIPP and ^18^F-FDG in EC-14 5 min after each addition. ^123^I-BMIPP accumulated in EC-14 at 6.65%ID/ng protein at 1 h of incubation, 3.52%ID/ng protein at 3 h and 2.68% ID/ng protein at 6 h. ^18^F-FDG accumulated in EC-14 at 6.32%ID/ng protein at 1 h of incubation, 15.9%ID/ng protein at 3 h and 11.8%ID/ng protein at 6 h. At a culture time of 1h, the accumulation of ^123^I-BMIPP was higher than that of ^18^F-FDG, whereas it was lower at culture times of 3 and 6 h. The accumulation of ^123^I-BMIPP in EC-14 5 min after injection and the growth curve of EC-14 are shown in Figure 1. ^123^I-BMIPP exhibited marked accumulation in *E. coli* EC-14 during the early growth phase.

### 3.2. Accumulation of ^123^I-BMIPP in E. coli EC-14 under Low-Temperature Conditions

**Figure 2:**
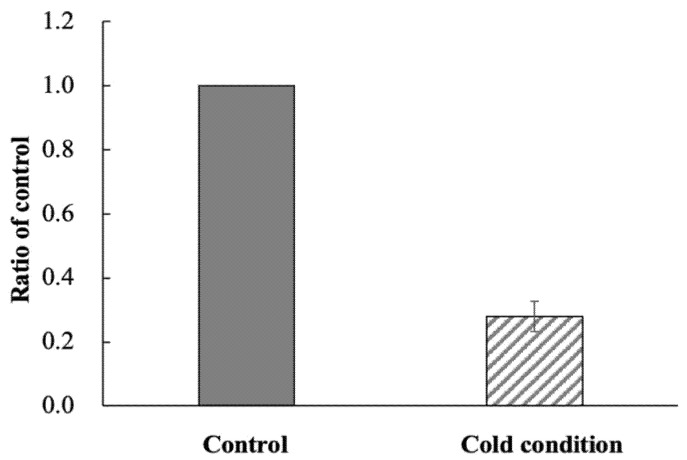
Accumulation of ^123^I-BMIPP in *E. coli* EC-14 under low-temperature conditions. EC-14 was incubated at 4 °C in amino-acid-free DMEM for 3 h. After incubation, ^123^I-BMIPP was added and incubated for 5 min at 4 °C. The accumulation rate of EC-14 at low temperature is significantly lower than that of the control at 37 °C.

The accumulation of ^123^I-BMIPP in *E. coli* EC-14 under low-temperature conditions is summarized in Figure 2. The accumulation rate was 0.28-fold lower than that of the control incubated at 37 °C.

### 3.3. Accumulation of ^123^I-BMIPP in E. coli EC-14 in the Presence of a CD36 Inhibitor

**Figure 3:**
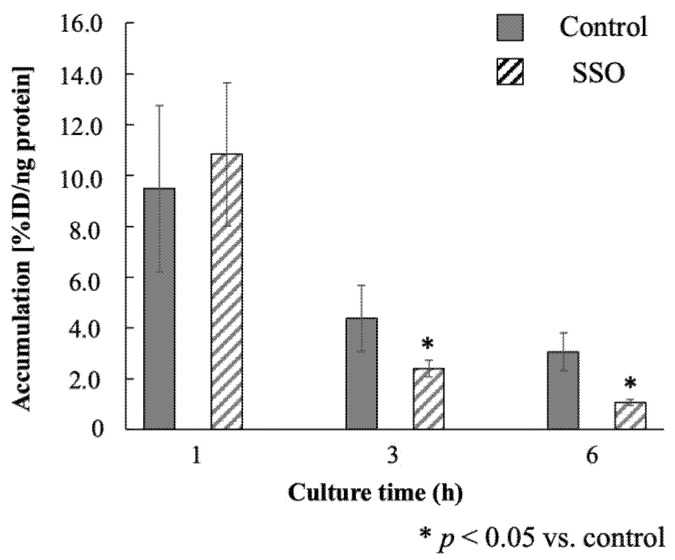
Accumulation of ^123^I-BMIPP in *E. coli* EC-14 in the presence of sulfosuccinimidyl oleate (SSO), a CD36 inhibitor. EC-14 was incubated at 4 °C in amino-acid-free DMEM for 1, 3 and 6 h. After incubation at 37 °C, 37 kBq/50 µL of ^123^I-BMIPP and 50 µL of 1.0 mM SSO (Cayman Chemical) were mixed and added. At 3 h and 6 h of incubation, there is a statistically significant decrease in ^123^I-BMIPP accumulation compared with control.

Figure 3 summarizes the accumulation of ^123^I-BMIPP in *E. coli* EC-14 incubated in the presence of a CD36 inhibitor. Under these conditions, ^123^I-BMIPP accumulated in EC-14 at 10.8%ID/ng protein at 1 h of incubation, 2.41%ID/ng protein at 3 h and 1.07%ID/ng protein at 6 h. The difference between the control and SSO treatment at 1 h of incubation was not significant. At 3 and 6 h of incubation, however, the rate of ^123^I-BMIPP accumulation was significantly lower than that of the control.

### 3.4. Growth of E. coli EC-14 in Infection Model Mice

**Figure 4:**
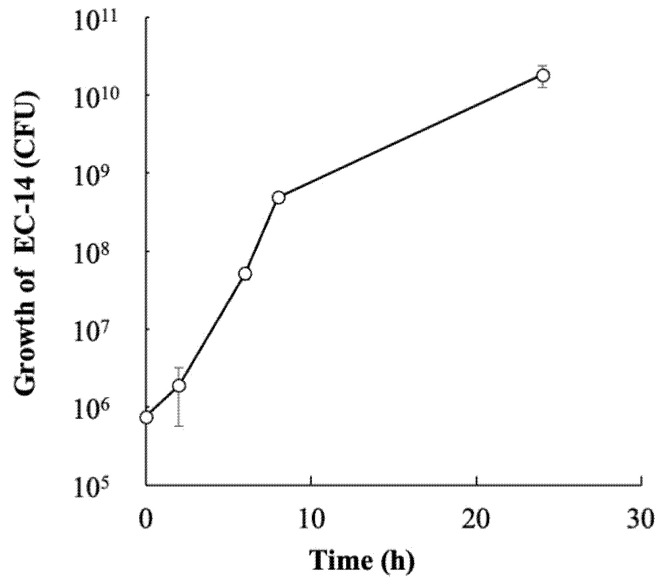
Growth curve of *E. coli* EC-14 in leg muscle of infection model mice. Immunosuppressed mice were infected with approximately 5 × 10^6^ CFU/100 µL of EC-14 in the muscle of the hind leg. At 2 h after infection, the mean number of EC-14 is approximately 1.9 × 10^6^ CFU, increasing to 4.9 × 10^8^ and 1.8 × 10^10^ CFU at 8 and 24 h after infection, respectively.

Figure 4 shows a growth curve for EC-14 in the leg muscle of model mice. On average, approximately 1.9 × 10^6^ CFU of EC-14 were present in the muscle tissue 2 h after infection, and the number of bacteria increased to 4.9 × 10^8^ and 1.8 × 10^10^ CFU at 8 and 24 h after infection, respectively.

### 3.5. Biological Distribution of ^123^I-BMIPP and ^18^F-FDG in EC-14 Infection Model Mice

Table 2 and Table 3 summarize the biological distribution of ^123^I-BMIPP and ^18^F-FDG, respectively, in EC-14 infection model mice. In comparison with the accumulation of ^18^F-FDG at 2 and 8 h after infection, the accumulation of ^123^I-BMIPP was higher in the blood, lung, liver and kidney.

Table 4 summarizes the accumulation of ^123^I-BMIPP and ^18^F-FDG in the EC-14-infected muscle and normal, uninfected muscle (control). The accumulation of both ^123^I-BMIPP and ^18^F-FDG was higher in the control mice than in the infected mice. ^123^I-BMIPP accumulation tended to be higher than that of ^18^F-FDG at both 2 and 8 h post-infection, and the rate of accumulation in the infected muscle was significantly higher 8 h post-infection. In this study, contrast means the ratio of the infected area to the contralateral normal area.

### 3.6. Planar Imaging with ^123^I-BMIPP in E. coli EC-14 Infection Model Mice

**Figure 5:**
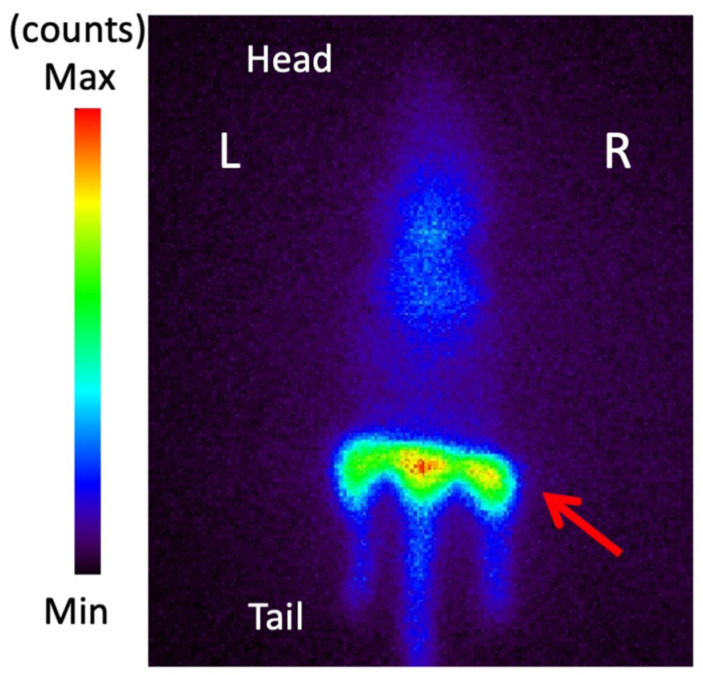
Imaging with ^123^I-BMIPP in *E. coli* EC-14 infection model mice. With lead shielding from head to bladder, planar images were acquired for 5 min (1 frame) at 1.5 h after ^123^I-BMIPP administration and 9.5 h after EC-14 infection. The infected muscle (arrow of right legs) is visualized, and the contrast is approximately 1.33 times higher than that of the control muscle (left legs).

Figure 5 shows the planar images acquired 1.5 h after ^123^I-BMIPP injection and 9.5 h after infection with *E. coli* EC-14. The infected area was visualized, and the contrast was approximately 1.33 times higher than that of the control area.

## 4. Discussion

In this study, we investigated bacterial imaging using a radiopharmaceutical in common clinical use for fatty acid metabolism scintigraphy. Since fatty acids are components of the bacterial cell membrane [9,10], we explored the use of ^123^I-BMIPP, a long-chain fatty acid analog used for myocardial fatty acid metabolism scintigraphy in Japan [11,12,13,14], for bacterial imaging. ^123^I-BMIPP exhibited marked accumulation in *E. coli* EC-14 during the early growth phase (Figure 1), similar to the accumulation of ^18^F-FDG (Table 1). This specific accumulation suggests that EC-14 actively metabolizes fatty acids. In addition, the accumulation of ^123^I-BMIPP in EC-14 was higher than that of ^18^F-FDG at the culture time of 1h, whereas it was lower at the culture times of 3 and 6 h because glucose concentration was regulated to 0.1 mg/mL in the culture medium for in vitro study with ^18^F-FDG, and the accumulation of ^18^F-FDG increased the most at the culture time of 3 h.

The mechanism of ^123^I-BMIPP accumulation in *E. coli* EC-14 was elucidated under the conditions of low temperature (Figure 2) and incubation in the presence of SSO, a CD36 inhibitor (Figure 3). In our group’s study, we have previously confirmed the inhibitory effect of SSO on the uptake of BMIPP in cancer cells (data not shown). Therefore, SSO was used in this study because it might also be relevant to bacterial accumulation. The rate of accumulation in EC-14 under low-temperature conditions was significantly lower than that of the control incubated at 37 °C, potentially due to a reduction in bacterial metabolic activity at the lower temperature, thereby impeding the uptake of ^123^I-BMIPP. This result indicates that the accumulation of ^123^I-BMIPP in EC-14 depends on both bacterial growth and metabolic activity. In the presence of SSO, an inhibitor of the fatty acid transport membrane protein CD36 [15,16], the accumulation of ^123^I-BMIPP was significantly reduced at 3 and 6 h of incubation compared with the control. This suggests that the mechanism of ^123^I-BMIPP uptake by EC-14 is sensitive to SSO and involves a fatty acid transport membrane protein such as CD36, which is present in human cells. CD36 binds long-chain fatty acids and promotes their transport into cells [17]. The lack of significance of the difference between the control and SSO treatment in the present study at 1 h of incubation was possibly due to a large measurement error. In addition, during the early stage of bacterial growth, other uptake mechanisms and/or metabolic activity could play a significant role in uptake. As the incubation time increased, the effect of SSO became more notable, which suggests that the bacterial uptake of ^123^I-BMIPP is sensitive to transporter selection. In addition, during the early stage of bacterial growth, other uptake mechanisms and/or metabolic activity could play a significant role in uptake. As the incubation time increased, the effect of SSO became more notable, which suggests that the bacterial uptake of ^123^I-BMIPP is sensitive to transporter selection. In addition, SSO inhibits the mitochondrial respiratory chain, and reduced mitochondrial activity may reduce ^123^I-BMIPP uptake. This is particularly important at later time points where significance is shown. The possibility that ^123^I-BMIPP is also taken up by bacteria through other pathways such as endocytosis cannot be excluded. However, we assume that the CD36-like uptake mechanism is at least one of the mechanisms of ^123^I-BMIPP uptake by bacteria.

Biological distribution was examined in the *E. coli* EC-14 infection model mice using ^123^I-BMIPP (Table 2) and ^18^F-FDG (Table 3). Compared with ^18^F-FDG, the accumulation of ^123^I-BMIPP in the blood, lung, liver, and kidney was higher at 2 and 8 h post-infection, indicating that ^123^I-BMIPP has a longer retention time than ^18^F-FDG in the primary organs. As for the blood clearance result, the lack of clearance from blood was likely affected by proteins in the mouse body. Studies have shown that serum albumin plays a role in binding and transporting fatty acids in the blood [18]. ^123^I-BMIPP, a derivative of fatty acid, binds to albumin in the mouse body, causing its retention in the blood. This may have resulted in the delayed excretion of ^123^I-BMIPP in this study. Moreover, in the in vivo experiment, the mice were fully anesthetized and not moving at about 2 h post-infection, but at 8 h post-infection, the mice were physically active, and the radioactivity in the blood was thought to have been transferred to the myocardium. An increased myocardial accumulation of ^18^F-FDG after exercise has been reported [19]. One can assume that this may have led to the increased ^18^F-FDG uptake in the heart. In addition, ^123^I-BMIPP accumulation was higher than that of ^18^F-FDG in non-infected control muscle (Table 4) because ^123^I-BMIPP may have had a greater impact on walking and/or running than ^18^F-FDG during the time between the infection and sacrifice of the mice. ^123^I-BMIPP exhibited a tendency toward greater imaging contrast at both 2 and 8 h after infection compared with ^18^F-FDG. A significant increase in accumulation in the infection area was observed at 8 h after infection (Table 4). Since ^123^I-BMIPP accumulation reportedly decreases in myocarditis [20], it can be inferred that ^123^I-BMIPP accumulated in the bacteria rather than in the inflamed tissues in this study.

^123^I-BMIPP imaging in the *E. coli* EC-14 infection model mice was performed with lead shielding from head to bladder (Figure 5). In this study, the right leg of the mouse was the *E. coli*-infected side, and the left leg was the non-infected side (control); the contrast indicates the ratio of the infected area to the contralateral normal area. Thus, the image of one mouse shows both infected and non-infected sides at the same time. The infected muscle imaged 1.5 h after ^123^I-BMIPP injection and 9.5 h after infection exhibited higher contrast than the non-infected control muscle. The infected area, as determined by the ^123^I-BMIPP signal, was approximately 1.33 times larger than the ^123^I-BMIPP control area. Analysis of the biodistribution of ^123^I-BMIPP 8 h after infection (Table 4) showed that the ratio of ^123^I-BMIPP accumulation in the infected muscle to that in control muscle was 1.29 at 15 min and 1.31 at 60 min after ^123^I-BMIPP injection. Thus, the lead shield had a negligible effect on planar imaging.

Clinical SPECT imaging in humans may show less ^123^I-BMIPP accumulation in control muscle than imaging in mice, because humans can rest before SPECT imaging. This may result in a higher image contrast at the infection site, making infected areas more clearly visible in clinical SPECT imaging.

As a limitation of this study, there may be difficulty when using ^123^I-BMIPP to detect bacterial infections in the trunk of the body, because of its accumulation and retention, but it could be very useful in infections involving the upper and lower limbs. ^123^I-BMIPP accumulates in normal muscle, but it has better contrast ratios than ^18^F-FDG at sites of bacterial infection (Table 4).

## 5. Conclusions

^123^I-BMIPP can take up *E. coli* EC-14 via a fatty acid transport membrane protein such as CD36 and accumulate by fatty acid metabolism. ^123^I-BMIPP has the potential to visualize fatty acid metabolism in bacteria for bacterial infection imaging, especially in upper and lower limbs.

## Figures and Tables

**Table 1:** Accumulation of radiopharmaceuticals in *E. coli* EC-14.

	Accumulation (%ID/ng Protein)	
Culture Time (h)	^123^I-BMIPP	^18^F-FDG (0.1 mg/mL Glucose Concentration)
1	6.65 ± 0.49	6.32 ± 2.31
3	3.52 ± 0.20	15.9 ± 1.37
6	2.68 ± 0.23	11.8 ± 0.49

%ID/ng protein indicates percent injected dose per ng of protein.

**Table 2:** Biological distribution of ^123^I-BMIPP in *E. coli* EC-14 infection model mice.

Accumulation of ^123^I-BMIPP (%ID/g)				
Time after Infection (h)	2		8	
Time after ^123^I-BMIPP Injection (min)	15	60	15	60
Blood	16.69 ± 0.93	16.10 ± 1.88	13.65 ± 3.57	19.43 ± 2.91
Heart	25.86 ± 6.46	29.11 ± 4.48	30.37 ± 3.90	30.66 ± 6.40
Lung	13.02 ± 2.00	11.84 ± 1.02	11.99 ± 1.10	12.01 ± 1.65
Liver	17.31 ± 3.86	11.04 ± 1.92	20.84 ± 3.32	12.02 ± 1.91
Kidney	14.01 ± 1.30	12.18 ± 1.27	12.00 ± 1.32	11.79 ± 1.88

%ID/g indicates percent injected dose per gram of tissue.

**Table 3:** Biological distribution of ^18^F-FDG in *E. coli* EC-14 infection model mice.

Accumulation of ^18^F-FDG (%ID/g)				
Time after Infection (h)	2		8	
Time after ^18^F-FDG Injection (min)	15	60	15	60
Blood	2.83 ± 0.90	0.69 ± 0.09	0.93 ± 0.11	0.18 ± 0.03
Heart	13.95 ± 5.47	15.05 ± 3.35	30.02 ± 7.33	28.57 ± 5.98
Lung	2.91 ± 0.76	2.96 ± 1.04	3.44 ± 0.39	4.04 ± 0.88
Liver	2.95 ± 1.07	1.10 ± 0.26	2.81 ± 0.49	2.10 ± 0.65
Kidney	5.43 ± 1.95	2.15 ± 0.52	5.77 ± 1.55	2.71 ± 0.67

%ID/g indicates percent injected dose per gram of tissue.

**Table 4:** Accumulation of ^123^I-BMIPP and ^18^F-FDG in *E. coli* EC-14 infection model mice.

After Infection (h)	After Injection (min)		^123^I-BMIPP		^18^F-FDG	
			Accumulation (%ID/g)	Contrast	Accumulation (%ID/g)	Contrast
2	15	Infected	6.09 ± 3.72	1.15	1.69 ± 0.50	1.05
		Control	5.30 ± 0.72		1.61 ± 0.53	
	60	Infected	6.13 ± 1.14	1.00	2.47 ± 0.69	0.97
		Control	6.10 ± 1.03		2.54 ± 0.81	
8	15	Infected	6.82 ± 2.02 *	1.29	4.25 ± 0.92	0.93
		Control	5.29 ± 0.63		4.58 ± 1.53	
	60	Infected	8.64 ± 1.80 *	1.31	3.14 ± 1.16	1.03
		Control	6.59 ± 0.80		3.05 ± 1.93	

%ID/g indicates percent injected dose per gram of tissue. * *p* < 0.01 vs. control muscle at about 8 h after infection.
